# Molecular Characterization of Dengue Virus Serotype 2 Cosmospolitan Genotype From 2015 Dengue Outbreak in Yunnan, China

**DOI:** 10.3389/fcimb.2018.00219

**Published:** 2018-06-27

**Authors:** Liming Jiang, Dehong Ma, Chao Ye, Lihua Li, Xiaoman Li, Jiajia Yang, Yujiao Zhao, Juemin Xi, Xiaodan Wang, Junying Chen, Yue Pan, Xiyun Shan, Qiangming Sun

**Affiliations:** ^1^Institute of Medical Biology, Chinese Academy of Medical Sciences and Peking Union Medical College, Kunming, China; ^2^Yunnan Key Laboratory of Vaccine Research and Development on Severe Infectious Diseases, Kunming, China; ^3^Yunnan Key Laboratory of Vector-borne Infectious Disease, Kunming, China; ^4^Xishuangbanna Dai Autonomous Prefecture People's Hospital, Xishuangbanna, China; ^5^School of Basic Medicine, Kunming Medical University, Kunming, China; ^6^The Affiliated Children's Hospital of Kunming Medical University, Kunming, China

**Keywords:** dengue virus, genome, characterization, phylogenetic analysis, recombinant analysis

## Abstract

In 2015, a dengue outbreak with 1,067 reported cases occurred in Xishuangbanna, a city in China that borders Burma and Laos. To characterize the virus, the complete genome sequence was obtained and phylogenetic, mutation, substitution and recombinant analyses were performed. DENV-NS1 positive serum samples were collected from dengue fever patients, and complete genome sequences were obtained through RT-qPCR from these serum samples. Phylogenetic trees were then constructed by maximum likelihood phylogeny test (MEGA7.0), followed by analysis of nucleotide mutation and amino acid substitution. The recombination events among DENVs were also analyzed by RDP4 package. The diversity analysis of secondary structure for translated viral proteins was also performed. The complete genome sequences of four amplified viruses (YNXJ10, YNXJ12, YNXJ13, and YNXJ16) were 10,742, 10,742, 10,741, and 10,734 nucleotides in length, and phylogenetic analysis classified the viruses as cosmopolitan genotype of DENV-2. All viruses were close to DENV Singapore 2013 (KX380828.1) and the DENV China 2013 (KF479233.1). In comparison to DENV-2SS (M29095), the total numbers of base substitutions were 712 nt (YNXJ10), 809 nt (YNXJ12), 772 nt (YNXJ13), and 841 nt (YNXJ16), resulting in 109, 171, 130, and 180 amino acid substitutions in translated regions, respectively. In addition, compared with KX380828.1, there were 44, 105, 64, and 116 amino acid substitutions in translated regions, respectively. The highest mutation rate occurred in the prM region, and the lowest mutation rate occurred in the NS4B region. Most of the recombination events occurred in the prM, E and NS2B/3 regions, which corresponded with the mutation frequency of the related portion. Secondary structure prediction within the 3,391 amino acids of DENV structural proteins showed there were 7 new possible nucleotide-binding sites and 6 lost sites compared to DENV-2SS. In addition, 41 distinct amino acid changes were found in the helix regions, although the distribution of the exposed and buried regions changed only slightly. Our findings may help to understand the intrinsic geographical relatedness of DENV-2 and contributes to the understanding of viral evolution and its impact on the epidemic potential and pathogenicity of DENV.

## Introduction

Dengue virus (DENV) belongs to the Flavivirus genus and is transmitted by *Aedes aegypti* and *Ae. Albopictus* mosquitoes, found in tropical and subtropical regions of world (Bhatt et al., 2013). DENV annually infects approximately 50 million people in more than 100 countries (maybe mention here that DENV can be lethal due to hemorrhagic fever or cite number of deaths—or the lack of effective vaccine or problem with antibody-dependent enhancement due to serotypes) (San Martín et al., 2010). The WHO declared that along with climate change, economic integration and migration have contributed to the expanded geographical range of DENV over the past decade (WHO, 2015).

There are four serotypes (DENV-1/2/3/4) that are closely related but are nonetheless antigenically and genetically distinct. Each DENV serotype is further subdivided into several phylogenetically distinct genotypes (Weaver and Vasilakis, 2009). The serotypes were identified by the difference of antigenicity and the genotypes were identified by the phylogenetic tree of DENV gene sequences. The genome of DENV is a linear, non-segmented, positive-sense strand of RNA of approximately 10.6–11 kb, and the MW is 4.2 × 10^6^ (Dash et al., 2015), and the full-length polyprotein which is processed by viral and host proteases into seven non-structural proteins (NS1, NS2A, NS2B, NS3, NS4A, NS4B, and NS5) and three structural proteins (capsid, premembrane, and envelope) (Holmes and Twiddy, 2003). The 3' UTR lacks a poly real (A) tail; they have A non-coding regions in 5′ end and 3′ end (Markoff, 2003). The four DENV serotypes share 65–70% sequence homology and are further clustered into different genotypes on account of high mutation rates (Holmes and Twiddy, 2003; Anoop et al., 2012). Each of the four serotypes of DENV (DEVN 1–4) can cause a spectrum of illness in human from mild dengue fever (DF) aggravate to severe life-threatening dengue shock syndrome (DSS) and dengue hemorrhagic fever (DHF) (Rodenhuis-Zybert et al., 2010).

Xishuangbanna (N22°0′42.00′′, E100°47′45.68′′) is located in the southernmost prefecture of Yunnan Province and is situated along a tropical rainforest area where dengue fever is endemic. A population of more than 1 million and the long summer without winter. Imported cases of DENV infection sporadically occur in bordering regions of Yunnan Province, such as Dehong and Xishuangbanna (Wang et al., 2015). The first outbreak of dengue fever in Yunnan was reported in 2008, with 56 confirmed cases (MOH, 2008). After the initial outbreak, larger epidemics have been regularly reported in Xishuangbannan. For instance, 1,538 infection cases were reported in 2013, 1,067 infection cases were reported in 2015, and 1,184 infected patients were detected by November 2017, indicating that dengue fever remains an epidemiological threat in Yunnan (Zhang et al., 2014; Wang et al., 2015, 2016; Yang et al., 2015; Zhao et al., 2016). A previous study by Zhao et al. found that the DENV-2 epidemic of Xishuangbanna in 2015 was most similar to the Indian and Sri Lankan epidemics that occurred in 2001 and 2004, respectively (Zhao et al., 2016).

In 2015, the first case of dengue fever in Xishuangbanna was reported on July 13th and the epidemic continued to 15th of November, with more than 1,000 confirmed cases. So far, detailed genomic characterization and identification of molecular recombination events during this DENV-2 outbreak have not been completed. In this article, we report for the first time the complete genomic sequences and comprehensive genetic analyses of four DENV-2 isolates from the 2015 outbreak in Yunnan, China. These findings supplement our understanding of flavivirus genetics and endemic transmission of DENV originating from the border areas of China, Laos, Burma, and Vietnam.

## Materials and methods

### Ethics statement

Ethical approval was obtained from the Institutional Ethics Committee (Institute of Medical Biology, Chinese Academy of Medical Sciences, and Peking Union Medical College). The study protocol was in accordance with the Declaration of Helsinki for Human Research of 1974 (last modified in 2000). Written informed consent was received from each patient before sample collection.

### Samples

During the dengue outbreak in the Xishuangbannan, Yunnan Province in 2015, the serum samples were collected from DENV-NS1 positive human patients at Xishuangbanna Dai Autonomous Prefecture People's Hospital (XDAPPH). A total of 852 DENV-NS1 positive serum samples were obtained. Sera of four patients were randomly selected for complete genomic analysis of DENV. The four patients ranged in age from 23 to 58 years old, without record of traveling abroad, and developed symptoms of fever, with fatigue and body rash.

### ELISA test

DENV IgG/IgM was detected in each sample using Dengue Virus IgG/IgM ELISA kit (Neobioscience Technology Co., Ltd., China). The assay was performed according to the operation manual.

### Virus RNA extraction, RT-PCR, and genomic sequencing

Serum samples were separated from collected blood. Viral RNA was extracted from 150 μL of collected serum using the RNA mini kit (Qiagen, Hilden, Germany) and eluted in 50 μL of nuclease-free water. The extracted RNA was used for RT-qPCR amplification, and genomic sequencing was carried out as previously described (Drosten et al., 2002). The One-step PrimeScriptTM RT-qPCR kit (TaKaRa Co., Ltd. Dalian, China) was used to amplify two overlapping fragments in the virus gene by RT-qPCR with the following protocol: initial reverse transcription at 42°C for 45 min; 35 cycles of denaturation at 94°C for 30 s, annealing at 55°C for 30 s, elongation at 72°C for 1 min and a final elongation step at 72°C for 5 min. Then, the PCR products were purified and sequenced by Sangon Biotech after identification with agarose gel electrophoresis (AGE).

The complete viral genomic sequences were sequenced in 22 fragments. The primer pairs were selected from Primer-BLAST in NCBI to amplify the DENV-2 genome based on the standard of M29095 (Irie et al., 1989). All primers were synthesized and purified through Sangon Biotech (Shanghai, China). A total of 22 overlapping amplifications spanning the complete genomic region were amplified using 44 primers (Table 1). The amplification of various genomic fragments was implemented following the standard methods (Wang et al., 2015). The specific PCR products were purified using the gel extraction kit (Qiagen, Germany) followed by double pass sequencing (Sangon Biotech, Shanghai, China). The 5′ 22 nucleotides and 3′ 23 nucleotides were obtained from the NCBI database.

**Table 1:** Primers used for the complete genomic characterization of DENV-2.

**Primer name**	**Sequence 5^′^-3^′^**	**Position**	**Length**
DV2-1-F	AGTTGTTAGTCTACGTGGACCG	1–22	656
DV2-1-R	GTGGACGTAGAGTTGCACCA	637–656	
DV2-2-F	ACCCTCATGGCCATAGACCT	541–560	557
DV2-2-R	GGCAGGTTGTTTGGCTTCTG	1079–1098	
DV2-3-F	GGGGTTTCAGGAGGAAGCTG	976–995	767
DV2-3-R	TTCTGTAGCCCCTGTGAGTG	1724–1743	
DV2-4-F	CCCACGCCAAGAAACAGGAT	1664–1683	538
DV2-4-R	AAATCCCAGGCTGTGTCACC	2182–2202	
DV2-5-F	AACCGGGACAACTGAAGCTC	2084–2103	457
DV2-5-R	TGAAGGGGATTCTGGTTGGA	2522–2541	
DV2-6-F	GGTGCAGGCTGATAGTGGTT	2412–2431	595
DV2-6-R	CATGGACGGCTCTGTTGTCT	2988–3007	
DV2-7-F	GCCCTGAAACAGCAGAATGC	2831–2850	735
DV2-7-R	TTCGTTCCTACTCGGGTCCT	3547–3566	
DV2-8-F	TCAACTCCTTGGTCACAGCC	3458–3477	528
DV2-8-R	GTGCAACTCACCTTCCATGC	3967–3986	
DV2-9-F	CTTTCCCAAAGCACCTTGCC	3808–3827	683
DV2-9-R	TGCTGCTGCCGTAATTGGTA	4472–4491	
DV2-10-F	AGGAAGCAGCCCAATTCTGT	4335–4354	565
DV2-10-R	ATACAGCGCCTATGGTTCCG	4881–4900	
DV2-11-F	ATCCCAGAGCTGTCCAAACG	4835–4854	743
DV2-11-R	CCGTGACCCATTCATGTCCT	5559–5578	
DV2-12-F	GGTGAAGCAGCCGGGATTTT	5440–5459	590
DV2-12-R	TCCTTCGGGTGTGTTGATGT	6011–6030	
DV2-13-F	CAGCGCAAAGAAGAGGGAGA	5882–5901	681
DV2-13-R	GCTGCCAGGAGTGTCAGTAG	6544–6563	
DV2-14-F	CTGCATACGGCTGAGGTAGG	6466–6485	693
DV2-14-R	GAAGAGCTGCTGTGAGGGTT	7140–7159	
DV2-15-F	ACAGCCATTGCTAACCAAGC	7021–7042	729
DV2-15-R	TTGCTGATCCTCGTGACACA	7750–7731	
DV2-16-F	TGCTGTGTCACGAGGATCAG	7728–7747	491
DV2-16-R	TCATGCGTGGAGTTTCGTGA	8200–8219	
DV2-17-F	CCCACGATAGAAGCAGGACG	8209–8048	346
DV2-17-R	GTCAGCAGTCTGACCACTCC	8536–8555	
DV2-18-F	CCACCCATACAAAACGTGGG	8460–8479	623
DV2-18-R	CTCAGGGAGTTCCCTCTGGA	9064–9083	
DV2-19-F	GGAGCTGGTTGACAGGGAAA	8865–8884	631
DV2-19-R	CAGCGATTTCTTCTGTGGCG	9477–9496	
DV2-20-F	AAGACCAAAGAGGCAGTGGG	9356–9375	578
DV2-20-R	GAACCCAATGTGACGGGACT	9915–9934	
DV2-21-F	ATGTACTTCCACAGACGCGA	9862–9881	510
DV2-21-R	TTTAACGTCCTTGGACGGGG	10353–10372	
DV2-22-F	TGCGGCTCATTGATTGGGTT	10108–10127	615
DV2-22-R	AGAACCTGTTGATTCAACAGCA	10701–10723	

### Genomic characterization and phylogenetic analysis

The 22 sequences were assembled using DNASTAR version 7.0. The assembled nucleotide sequences and translated amino acid sequences were analyzed by BioEdit. Phylogenetic analysis, based on the complete genomes, was conducted using the Molecular Evolutionary Genetics Analysis (MEGA) software version 7.0 (maximum likelihood phylogeny test) and gamma-distributed rates among sites with 1,000 bootstrap replicates.

The reference DENV-2 complete viral genome sequences used to construct the distinct phylogenetic branches were obtained from the GenBank sequence database under the following country and accession numbers: China (KC131142.1, EU359009.1, KF479233.1, JX470186.1); Philippines (KU517847.1, KU509276.1); Australia (KX372564.1, AY037116.1); Singapore (JN851127.1, KX380828.1); Indonesia Jakarta (Y858035.2); Malaysia Johor Bahru (KU666945.1); China Taiwan (DQ645552.1); Indonesia (GQ398258.1); Burkina Faso (EU056810.1); India Hyderabad (JX475906.1); Djibouti or Ethiopia (LC121816.1); Saudi Arabia (KJ830750.1); Sri Lanka (GQ252676.1); India (DQ448231.2); Papua New Guinea (KM204118.1); standard (M29095, KU725663.1); USA (JF730054.1, KM587709.1, EU687243.1); Thailand (FJ810410.1, GQ868542.1); Viet Nam (FJ461305.1, JX649148.1); Peru Puerto Maldonado (KC294201.1); Venezuela Aragua (GQ868541.1); Puerto Rico (GQ398314.1); Haiti (KY415992.1); Brazil (KP188555.1); Guadeloupe (EU920850.1); Colombia Guaviare (FJ182012.1); Venezuela HQ332190.1); Venezuela Aragua (FJ639822.1); Mexico (KJ189370.1); Nicaragua Managua (EU482680.1); South Korea (KP406804.1); Tonga (HM582115.1).

### Recombination analysis

Recombination and molecular evolution analysis was conducted with RDP4.56 package (Martin et al., 2010). The reference viral sequences used in the recombination analysis were obtained from the GenBank sequence database based on phylogenetic trees or geographically close viral sequences under the following accession numbers: KX380828.1-2013-Singapore; KF479233.1-2013-China; JX475906.1-2009-India, Hyderabad; JN851127.1-2004-Singapore; KX372564.1-2015-Australia; KP188555.1-2013-Brazil; EU687243.1-2004-USA, Puerto Rico.

### Secondary structure analysis of complete genome

PredictProtein (https://www.predictprotein.org/) was used to calculate the differences in the secondary structure between the structural and non-structural proteins of the DENV-2SS and 2015 Xishuangbanna epidemic viruses. The amino acid composition and potential RNA, DNA, nucleotide and protein binding sites were analyzed. The potential helical structure was also evaluated.

## Results

### Laboratory diagnosis

All 4 patients had typical dengue-like symptoms, including headache, fever, joint pain, myalgia, vascular leakage, pleural effusion, vomiting and nausea. Laboratory investigations of the patients revealed low platelet counts (<100 ^*^ 10^9^/L) and elevated liver enzyme levels (>100 U/L) (including alanine amino transferase and aspartate amino transferase). Further analysis showed that patients' sera were positive for anti-dengue IgM antibodies but tested negative for anti-dengue IgG antibodies, indicating an acute primary dengue infection. After a week of hospitalization, the four patients recovered and then were discharged.

### Nucleotide sequence analysis of the YNXJ10, YNXJ12, YNXJ13, and YNXJ16 sequences

RT-PCR amplification generated 22 specific amplicons, which were gel purified and sequenced. After editing and aligning the sequences of overlapping fragments, the complete genome sequences of the newly obtained viruses (named YNXJ10, YNXJ12, YNXJ13, and YNXJ16) were determined. The complete genome sequences of YNXJ10 and YNXJ12 were 10,742 nucleotides (nt) in length, with the ORF located between nucleotides 100–10,275. The length of YNXJ13 and YNXJ16 were 10,741 and 10,734 nt, respectively, with the ORF located between nucleotides 97–10,272 and 90–10,265, respectively.

The nucleotide composition of the four viruses were as follows: YNXJ10 (33.03% A, 25.41% G, 21.05% U, and 20.51% C), YNXJ12 (32.88% A, 25.48% G, 21.03% U, and 20.61% C), YNXJ13 (33.01% A, 25.40% G, 21.13% U, and 20.45% C), YNXJ16 (33.35% A, 25.72% G, 20.90% U, and 20.03% C). In comparison, the nucleotide composition of the DENV-2SS (M29095) virus was 33.09% A, 25.36% G, 20.88% U, and 20.66% C. The A+G content in YNXJ10, YNXJ12, YNXJ13, and YNXJ16 was similar to that of DENV-2 standard virus M29095 (58.45%). The complete genomic sequences of these four viruses were deposited into the NCBI GenBank database (https://www.ncbi.nlm.nih.gov/nuccore): KY937185, KY937187, KY937188, and KY937189.

### Genome phylogenetic analysis of the YNXJ10, YNXJ12, YNXJ13, and YNXJ16 sequences

**Figure 1:**
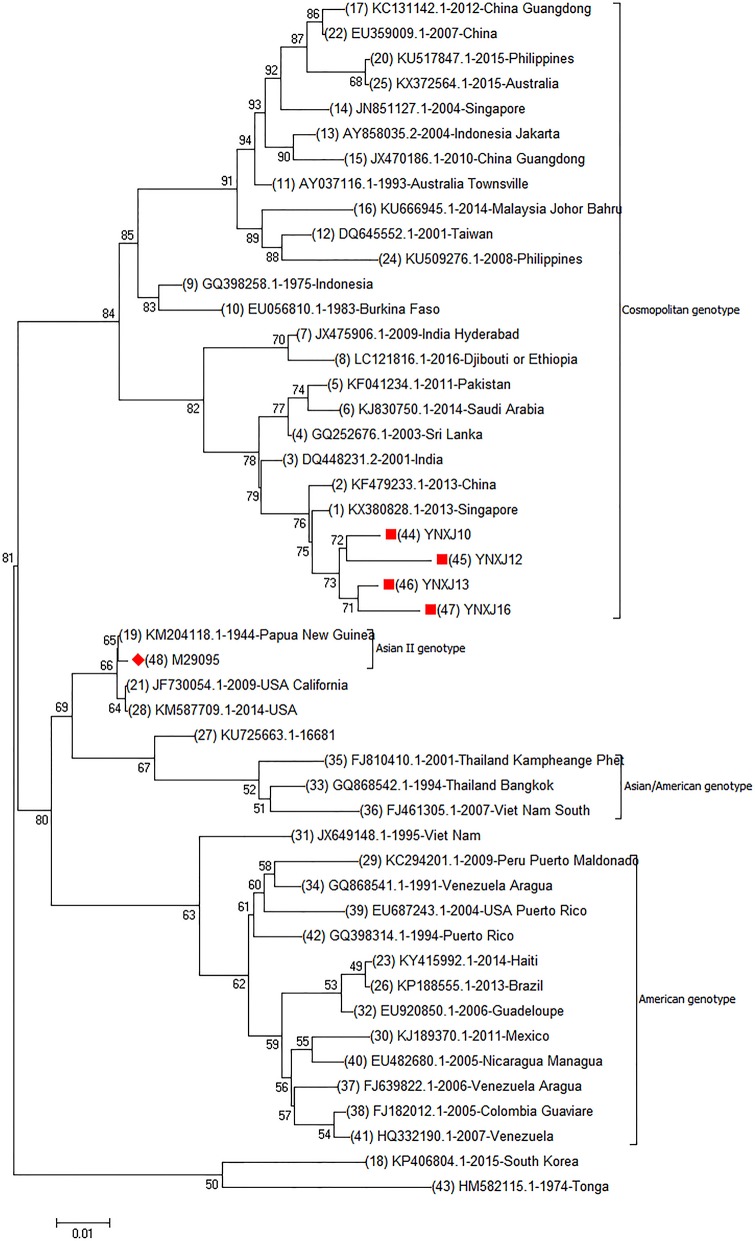
Complete genomic phylogenetic analysis of YNXJ10, YNXJ12, YNXJ13, and YNXJ16. Study sequences are labeled in the red circle. The DENV-2 standard M29095 are labeled with red diamond. Others are representative of DENV-2 from diverse geographical origins retrieved from GenBank. Phylogenetic analysis, based on the complete genomes, was conducted using the MEGA software version 7.0 (maximum likelihood phylogeny test) and gamma-distributed rates among sites with 1,000 bootstrap replicates.

Genome phylogenetic analysis of YNXJ10, YNXJ12, YNXJ13, and YNXJ16 were performed by aligning these viruses against 43 other representative DENV-2 viruses of diverse geographical origins retrieved from GenBank. The result indicated that the YNXJ10, YNXJ12, YNXJ13, and YNXJ16 viruses clustered in the cosmopolitan genotype close to DENV-2 KX380828.1-2013-Singapore and KF479233.1-2013-China. Not surprisingly, the closely related DENV-2 viruses from the 2015 epidemic, Philippines-2015 (KU517847.1) and Australia-2015 (KX372564.1), were from geographically near locations, and both of them belonged to the Asian genotype. Meanwhile, according to the phylogenetic analysis, the causative DENV-2 of the 2015 dengue outbreak in Yunnan most likely originated from the Singapore-2013(KX380828.1) virus, not from the Philippines-2015 (KU517847.1) or the Australia-2015 (KX372564.1) viruses (Figure 1).

### Base substitution and amino acid mutation analysis of the YNXJ10, YNXJ12, YNXJ13, and YNXJ16 coding sequences (CDS)

The Blastx-translated nucleotide sequence identity showed that YNXJ10, YNXJ12, YNXJ13, and YNXJ16 were highly matched, with an identity of 97 ~ 98%. However, the identity with the standard viruses DENV-2SS (m29095) was 91 ~92%.

The total number of amino acids in YNXJ10, YNXJ12, YNXJ13, and YNXJ16 was 3,392. As shown in Table 1, compared to standard viruses DENV-2SS (GenBank ID: M29095), the total numbers of base substitutions in YNXJ10, YNXJ12, YNXJ13, and YNXJ16 were 712, 809, 772, and 841 nt, respectively. The highest mutation rate was located at the coding region of structural protein prM, whereas the lowest mutation rate was found within the coding region of the non-structural protein NS4B.

The numbers of non-synonymous substitutions in YNXJ10, YNXJ12, YNXJ13, and YNXJ16 viruses were 327, 316, 383, and 301 nt, corresponding to rates of 44, 39, 49, and 36% of all the mutations, respectively.

#### Amino acid mutations in structural protein regions

In the structural protein regions of the four viruses, the nucleotide sequence coding for C-prM/M-E was 2,322 nt in length and codes for a 774 amino acid sequence. The lengths of the capsid, premembrane, and envelope amino acid sequences in the four viruses were 113, 166, and 495, respectively. Compared to the DENV-2 standard virus M29095, the total numbers of base substitution mutations in the C-prM-E region were 171 (YNXJ10), 148 (YNXJ12), 205 (YNXJ13), and 241 (YNXJ16), and the numbers of non-synonymous substitutions were 41 (YNXJ10), 26 (YNXJ12), 58 (YNXJ13), and 99 (YNXJ16). Consequently, the rate of non-synonymous mutations in the structural protein regions of the C-prM-E proteins were 5.30, 3.36, 7.50, and 11.50%, respectively (Table 2).

**Table 2:** Amino acid substitutions in the translated regions.

		**C**	**prM**	**E**	**NS1**	**NS2A**	**NS2B**	**NS3**	**NS4A**	**NS4B**	**NS5**	**Total**
YNXJ10 vs. M29095	Base substitution	20	49	102	69	54	29	142	61	18	168	712
	Base substitution rate	5.90	9.84	6.87	6.53	8.26	7.44	7.66	7.11	5.36	6.21	6.70
	AA substitutions	2	14	25	7	9	5	16	7	0	24	109
	AA substitution rate	1.77	8.43	5.05	1.99	4.13	3.85	2.59	2.45	0	2.66	3.21
YNXJ12 vs. M29095	Base substitution	20	33	95	71	54	32	179	99	18	208	809
	Base substitution rate	5.90	6.63	6.40	6.72	8.26	8.21	9.65	11.54	5.36	7.69	7.95
	AA substitutions	2	6	18	7	9	7	44	39	0	39	171
	AA substitution rate	1.77	3.61	3.63	1.99	4.13	5.38	7.12	13.64	0	4.32	5.04
YNXJ13 vs. M29095	Base substitution	21	94	90	76	52	22	138	69	18	192	772
	Base substitution rate	6.19	18.88	6.06	7.20	7.95	5.64	7.44	8.04	5.36	7.10	7.59
	AA substitutions	2	38	18	10	9	1	15	8	0	29	130
	AA substitution rate	1.77	22.90	3.63	2.84	4.13	0.77	2.43	2.80	0	3.22	3.83
YNXJ16 vs. M29095	Base substitution	51	62	128	75	54	22	175	68	18	188	841
	Base substitution rate	15.04	12.45	8.62	7.10	8.26	5.64	9.44	7.93	5.36	6.95	8.26
	AA substitutions	24	23	42	9	9	1	36	8	0	28	180
	AA substitution rate	21.2	13.86	8.48	2.56	4.13	0.77	5.83	2.80	0	3.10	5.31

**Figure 2:**
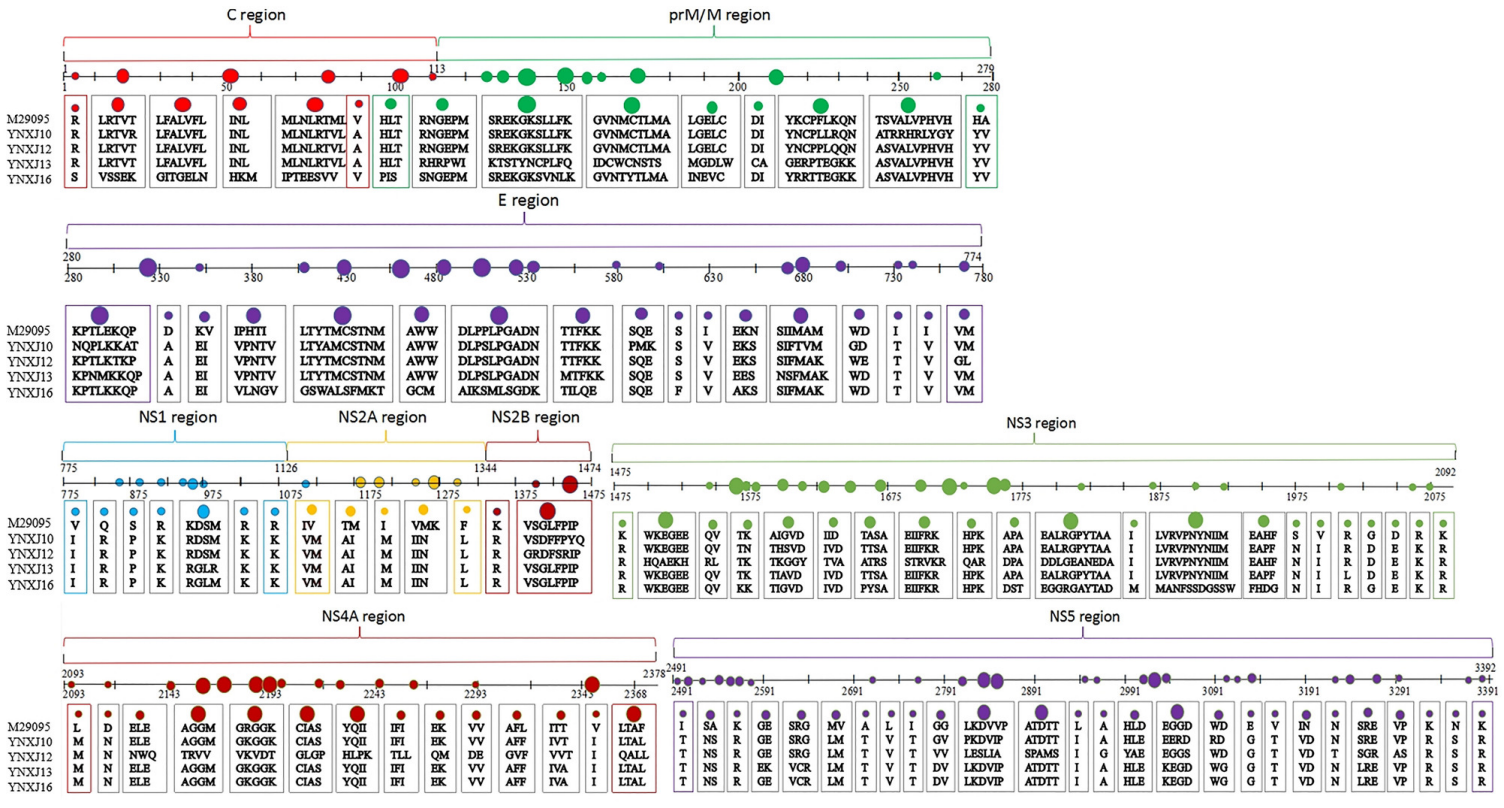
Amino acid substitutions in YNXJ10, YNXJ12, YNXJ13, and YNXJ16 compared to the DEN2SS M29095. The first line is M29095, and the second to fifth lines are YNXJ10, YNXJ12, YNXJ13, and YNXJ16, respectively. The labeled numbers represent the amino acid position.

Compared with M29095, three non-synonymous mutations were observed in the sequences encoding the β-sheet (196:T → A; 262:H → Y; 266:A → V), and 6 non-synonymous mutations were observed in the sequences encoding the coil (351:D → A; 406:K → E; 409:V → I; 421:I → V; 429:H → N; 444:I → V; 499:P → S), compared with M29095. The detected mutations are summarized in Figure 2.

As Figure 2 indicates, within Domain II of the E protein, a T to C substitution at position 1606 changes the amino acid S (Serine) to P (Proline) (amino acid position 536), which changes the polarity. Meanwhile, a G to A substitution at position 1612 was observed, and this mutation converted the negatively-charged amino acid E (Glutamic acid) to a positively-charged K (Lysine) (amino acid position 538).

#### Amino acid mutations in non-structural protein regions

Within the non-structural protein region, the length of the NS1-NS2A-NS2B-NS3-NS4A-NS4B-NS5 sequence of the four viruses was 7,854 nt. Compared with M29095, there were 69–76 single nucleotide changes identified in the NS1 region, including 7–10 non-synonymous substitutions (Figure 2). The base substitution T2710C modified the non-polar amino acid I (Isoleucine) to polar T (Threonine). At amino acid position 1266, K (Lysine) changed to N (Asparagine), which converted a basic amino acid to an uncharged amino acid in the coil.

**Figure 3:**
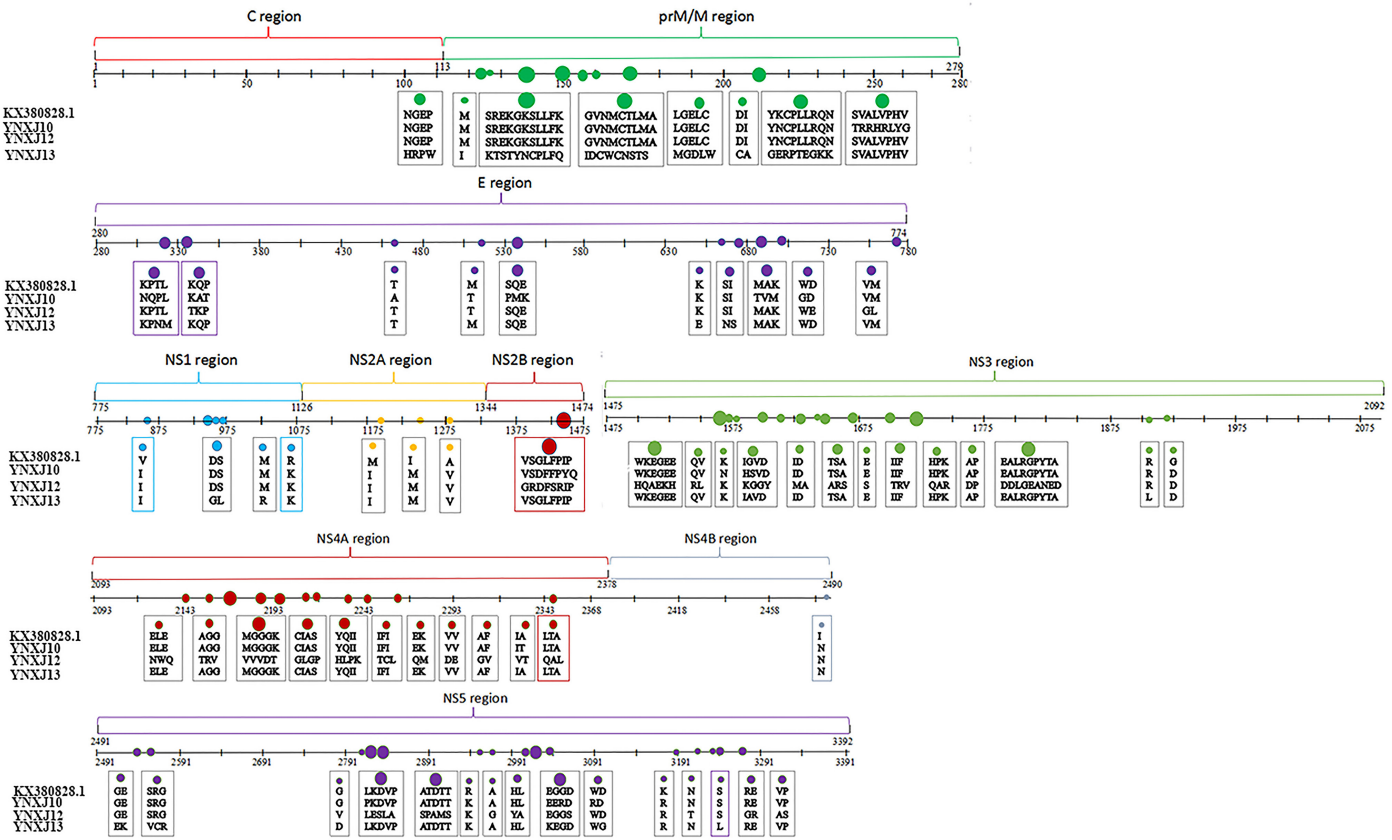
Amino acid substitutions of YNXJ10, YNXJ12, and YNXJ13 compared to the closely-related Singapore in 2013 (KX380828.1). The first line is KX380828.1, and the second to fourth lines are YNXJ10, YNXJ12, YNXJ13, respectively. The labeled numbers represent the amino acid position.

Compared with M29095, there were 76–86 base mutations found in the NS2A-NS2B region, and 10–16 were non-synonymous substitutions; the total number of base substitution mutations in the NS3 region was 138–179, and the number of non-synonymous substitutions was 16–44; (Figure 2). There were 7 and 39 amino acid substitutions in NS4A and NS4B, respectively. For NS5, the total number of base substitutions was 168-208, and there were 24–29 non-synonymous substitutions found in this region, with a substitution rate of 2.66 ~ 4.32% (Figure 2). In addition, compared with KX380828.1, there were 44, 105, and 64 amino acid substitutions in the translated regions of YNXJ10, YNXJ12, and YNXJ13, respectively (Figure 3).

### Recombination events of DENV-2 genome

**Figure 4:**
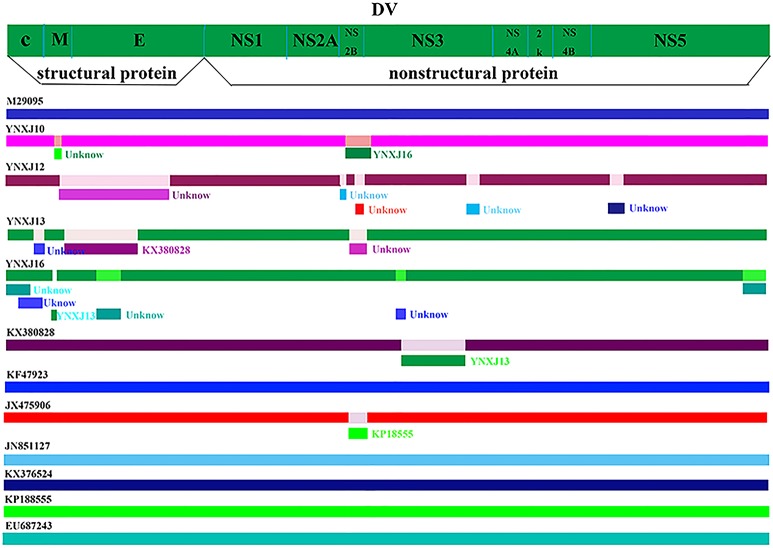
Recombination events in DENV-2. Schematic representation indicates the predictive recombination events among M29095, YNXJ10, YNXJ12, YNXJ13, YNXJ16, KX380828.1, KF479233, JX475906, JN851127, KX372564, KP188555, and EU687243, and analyzed by RDP package. Continuous lines indicate no recombination possibility.

The predictive complete genomic mutation map was performed in comparison with the closely related viruses, Singapore 2013 (KX380828.1), China 2013 (KF479233.1), and India 2009 (JX475906.1). Some recombination events may have occurred between the four viruses from Xishuangbanna and the closely related viruses, KX380828.1-2013-Singapore and KF479233.1-2013-China. There were many suspected recombination mutation areas in the complete genome. The suspected recombination mutations of the YNXJ10 virus might related to YNXJ16, while the suspected recombination mutations of YNXJ13 virus might related to KX380828. Meanwhile, the prediction results showed that the most likely recombination events were located at the structural genes prM and E, and no recombination events were observed in the non-structural region of 2K and NS4B (Figure 4).

### Possible secondary structures in structural and non-structural protein regions

The possible secondary structure of structural and non-structural proteins of YNXJ10, YNXJ12, YNXJ13, and YNXJ16 were then compared with that of M29095 (DEN2SS). Of the 3,391 total amino acids that code for the YNXJ structural and non-structural proteins, there are 10 amino acids that may participate in nucleotide binding. Seven possible nucleotide-binding sites (including 4, 10, 11, 13, 22, 67, and 82) were present only in YNXJ10, YNXJ12, YNXJ13, and YNXJ16, whereas the other three sites (6, 9, and 25) were lost, compared with M29095.

Forty-one changes at possible protein binding sites were observed among the 3,391 amino acids that form YNXJ structural and non-structural proteins. Of these, 24 possible protein binding sites (including 1, 4,94, 97, 98, 142, 158, 183, 236, 408, 663, 972, 1,156, 1,265, 1,582, 1,631, 1,935, 2,068, 2,084, 2,108, 2,377, 2,760, 2,863, and 2,880) were observed in YNXJ10, YNXJ12, YNXJ13, and YNXJ16, whereas sites 20, 26, 27, 28, 262, 429, 876, 1,131, 1,268, 1,587, 1,627, 1,915, 2,069, 2,080, 2,097, 2,269, and 2,862 were lost, compared with M29095.

**Figure 5:**
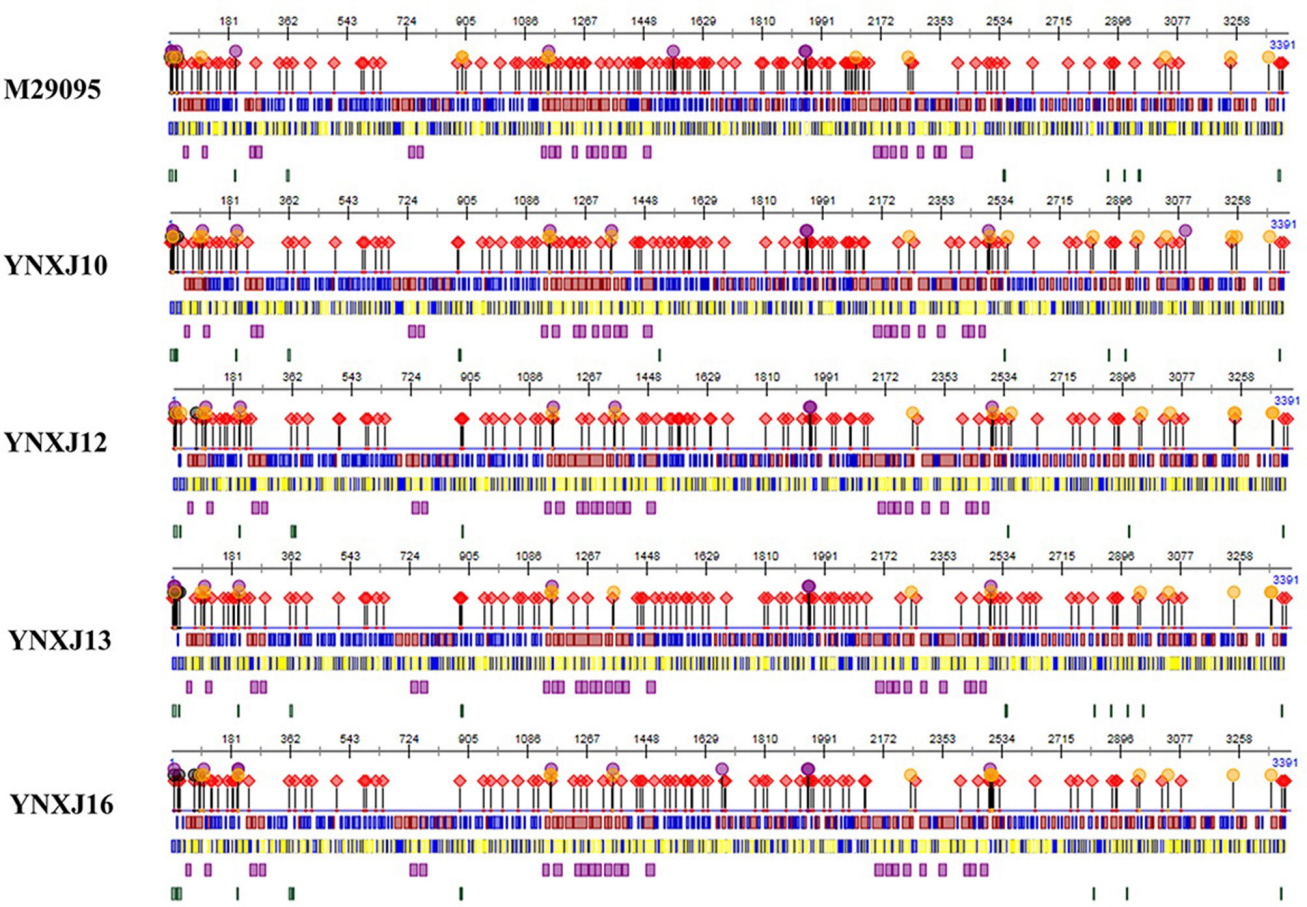
Secondary structure prediction of the structural and non-structural proteins for DEN2SS M29095 and YNXJ10, YNXJ12, YNXJ13, and YNXJ16. The purple dots denote the RNA-binding region, the black dots denote the nucleotide-binding region, the red rhombuses denote the protein-binding region, and the yellow dots denote the DNA-binding region. Red and blue in the first line represent the strand and helix regions, respectively. Yellow and blue in the second line represent the buried and exposed regions, respectively. Purple in the third line indicates the helical transmembrane regions, and green in the fourth line represents the disordered regions. The first map is M29095, and the second, third, fourth, and fifth maps are YNXJ10, YNXJ12, YNXJ13, and YNXJ16.

Approximately 41 distinct nucleotide mutations were observed in the helix regions, whereas only a few mutations were found in the buried and exposed regions. Moreover, there were 13 nucleotide mutations observed in the disordered region of YNXJ10, YNXJ12, YNXJ13, and YNXJ16 (Figure 5). Of these, 8 mutations (13, 15, 17, 201, 877, 880, 1,487, and 2,909) occurred in YNXJ10, YNXJ12, YNXJ13, and YNXJ16, while 5 mutations (including 358, 2,954, 3,382, 3,383, and 3,384) existed only in M29095.

## Discussion

The occurrence of dengue fever has increased remarkably in China in recent decades due to urbanization, globalization, climate change, migration and other factors (Murray et al., 2013; Chen and Liu, 2015; Guzman and Harris, 2015). The epidemic area, Xishuangbanna, Yunnan is located in southwestern China where dengue fever has been prevalent since 2008 (MOH, 2008). Since then, epidemics have been regularly reported in Xishuangbanna, Yunnan. A serious outbreak of DENV-3 occurred in 2013, with 1,538 infected individuals (Zhang et al., 2014; Wang et al., 2015, 2016; Yang et al., 2015). In 2015, Xishuangbanna experienced a large DENV-2 outbreak, which was the largest dengue epidemic in the past few years. Although the cause of this outbreak is not clear, it is coincident with the increasing global trend (Qin and Shi, 2014; Zhao et al., 2016). In recent years, the incidence of dengue fever in China's neighboring countries, such as Indonesia, Myanmar, Singapore and Malaysia, has been higher than in previous years (Dash et al., 2013; Ng et al., 2013). Xishuangbanna has close contact with Laos, Thailand and Myanmar. Furthermore, Xishuangbanna is a tourist destination and attracts more than 14 million tourists from around the world annually, resulting in an increased risk of DENV epidemic (Lowe et al., 2014).

The molecular characterization of DENV-2 at the genomic level is very important to understand the spread of dengue fever in Xishuangbanna. In this article, we were interested in extending previous studies and elucidating the genetic relationship between circulating DENV-2 viruses in southwest China and other parts of the world. Phylogenetic analysis and sequence alignment of the full-length genomes of YNXJ10, YNXJ12, YNXJ13, and YNXJ16 showed a close relationship with KX380828.1-2013-Singapore and KF479233.1-2013-China that are clustered in the Asian genotype.

Comparing the four Xishuangbanna DENV-2 sequences to KX380828.1-2013-Singapore and KF479233.1-2013-China, the greatest number of mutations occurred in the structural protein gene prM, while no mutation was observed in the non-structural gene NS4B. More mutations occurred in structural genes than in non-structural genes, indicating that structural genes are more variable while the non-structural genes are more stable under selective pressure. During host-pathogen interaction, the structural protein is located in the envelope region that interacts with host cell surface, which is under more selective pressure; however, the non-structural protein is located in the interior of the virion, which allows for minimal adaption from the host. This phenomenon coincided with the mutation patterns of YNXJ10, YNXJ12, YNXJ13, and YNXJ16.

The emergence of recombinant viruses could have a great impact on epidemiological and clinical outcomes. Interestingly, recombination events were observed between YNXJ13 and the KX380828.1-2013-Singapore. According to our prediction of possible recombination events, most recombination events were predicted to occur in the structural gene prM/E and non-structural gene NS2B/NS3. NS2B/NS3 helps the virus escape from the host immune system by cutting antiviral protein STING. As a protease, NS2B/NS3 also plays an essential role during flaviviral polyprotein processing. Thus, amino acid substitution in both prM/E and NS2B/NS3 proteins may greatly affect the efficiency of viral replication.

In summary, we report the first complete genome sequences of DENV-2 from Xishuangbanna, Yunnan, China. There were extensive outbreaks of dengue virus of different serotypes and genotypes in surrounding areas, such as Singapore, Taiwan, Guangdong, Vietnam, Burma, and Laos, in 2015. Hence, the origin of the Xishuangbanna epidemic is difficult to pinpoint with certainty. This study could help identify the role of geography and human migratory patterns that ultimately act in concert with intrinsic viral adaptive capabilities to result in large-scale outbreaks, and could offer further insight into DENV-2 pathogenicity, infectivity, and vaccine development.

## Author contributions

LJ, CY, JY, and XL: acquisition and analysis of data; drafting of the manuscript; DM, XS, and LL: providing blood samples and experimental requirement; YZ, JX, XW, JC, and YP: material or technical support; QS and XS: study concept and design; critical revision of the manuscript; study supervision; obtained funding. All authors read and approved the final manuscript.

### Conflict of interest statement

The authors declare that the research was conducted in the absence of any commercial or financial relationships that could be construed as a potential conflict of interest.
